# Venous waterfall and venous congestion

**DOI:** 10.1186/s40635-026-00879-4

**Published:** 2026-03-05

**Authors:** Jon-Emile S. Kenny, Per Werner Moller

**Affiliations:** 1 Health Sciences North Research Institute, Sudbury, ON Canada; 2 Flosonics Medical, Toronto, ON Canada; 3 Department of Anesthesia, SV Hospital Group, Institute of Clinical Sciences at the Sahlgrenska Academy, University of Gothenburg, Gothenburg, Sweden

Castro and colleagues have published a theoretical exploration of venous vascular waterfalls and venous congestion [1]. They describe how the ‘vascular waterfall’ dissociates downstream pressure (e.g., right atrial pressure, P_RA_) from blood flow (e.g., venous drainage). Waterfalls occur when the transmural pressure of a collapsible tube approaches zero and vessel collapse generates a ‘choke point,’ or ‘Starling resistor.’ In this setting, flow is proportional to the gradient between the upstream pressure (the mean systemic filling pressure P_MSF_) and the critical closing pressure (P_CRIT_) at the collapse point. Change in downstream pressure below P_CRIT_ does not affect flow. The authors note that venous Starling resistors occur within some visceral venous beds (e.g., intestine and liver), but not others (e.g., kidney). Given this, they argue that venous waterfalls ‘protect’ organs such as the liver and intestine from elevated P_RA_.

However, would not venous waterfall inherently limit venous drainage from the organ—as collapsing airways limit airflow from ‘congested’ alveoli? In obstructive airway disease, P_CRIT_ above airway pressure leads to gas trapping. Reducing P_CRIT_ relative to airway pressure (e.g., positive end-expiratory pressure) abolishes the airway Starling resistor with increasing airflow and diminished dynamic hyperinflation; there is rising flow and augmented downstream pressure. This is also observed in venous vascular beds. Experimentally increasing pulmonary venous pressure transitions West Zone II into West Zone III, increasing pulmonary blood flow. During inspiratory hold maneuvers, rising P_RA_ reduces hepato-splanchnic venous Starling resistors and venous resistance leading to increased hepatic venous outflow—but only in euvolemia [2]. The dynamic mechanism of vascular waterfalls, modulated by volume state, is one of several reasons why inspiratory hold maneuvers cannot be used to estimate P_MSF_ [3, 4].

These observations lead to a paradox. We accept that rising P_RA_ and flow is clinically common, but does rising downstream pressure (e.g., P_RA_) coupled with increasing flow constitute congestion? When a fluid-responsive patient receives preload, P_MSF_, venous return driving pressure, and flow all increase. We, therefore, agree with Guyton: “… *P*_*RA*_* is not one of the primary determinants of cardiac output but, instead, is itself determined simultaneously along with cardiac output*.” With Guyton’s perspective, P_RA_ is linked to venous congestion but does not cause it; congestion occurs when the independent variables of the circulatory system reduce flow and elevate* P*_*RA*_.

The ‘Geometrical model’ describes a system of simple equations that concurrently approximate blood flow (Q_CIRCULATORY_) and P_RA_ as functions of the circulation’s independent variables [5]. Per this model, when there is a venous Starling resistor, P_RA_ and Q_CIRCULATORY_ are:

1 $${P}_{RA}= \left[{(P}_{MSF}- {P}_{CRIT})\frac{{ R}_{CARDIAC}}{{R}_{VR}}\right]+ {P}_{PC}$$

2 $${Q}_{CIRCULATORY}= \frac{{P}_{MSF}- {P}_{CRIT}}{{R}_{VR}}$$

From this view, reducing P_CRIT_ (i.e., abolishing waterfall) increases P_RA_ and Q_CIRCULATORY_ all else equal (Fig. 1A). From the organ perspective, this is ‘decongestion’ akin to reduced dynamic hyperinflation described above. Equations 1 and 2 also reveal that with venous Starling resistors, there is no independent variable that causes P_RA_ to rise and flow to fall (i.e., ‘congestion’). Increasing pericardial pressure (P_PC_) and/or cardiac resistance (R_CARDIAC_) raises P_RA_ but does not decrease flow (Fig. 1B).

**Fig. 1:**
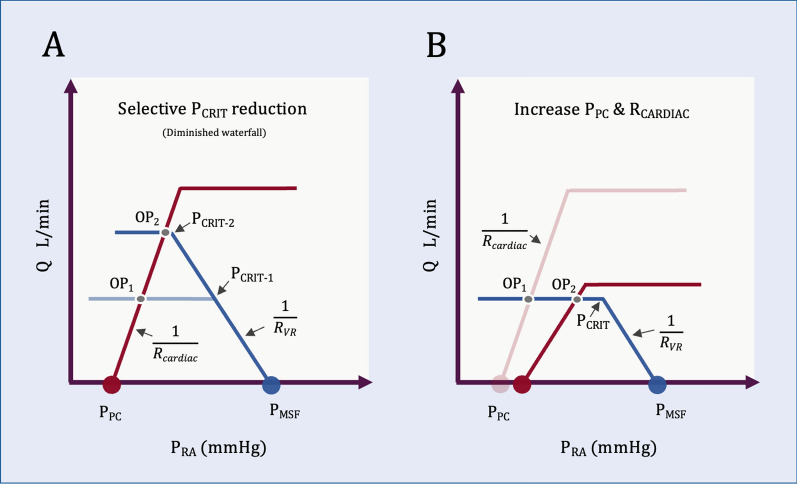
Venous waterfall and the Geometrical model. The blue curve is venous return; the red curve is cardiac function. **A** A venous waterfall reduces P_RA_, but this is not ‘protective,’ because the waterfall does so at the expense of reduced flow shown at operating point 1 (OP_1_). If venous waterfall is abolished by lowering P_CRIT_, OP_1_ moves to OP_2_, P_RA_ increases, but so too does flow out of the organ. **B** Variables that increase P_RA_ do not decrease flow with a venous waterfall. Rising P_PC_ (right shift of red curve) illustrates partial effect of increased airway pressure transmitted to the pericardial space. If ‘venous congestion’ is defined as the combination of rising P_RA_ and falling flow, venous Starling resistors do not allow this from a simple geometrical perspective

Taken together, a venous waterfall reduces P_RA_, but this is not ‘protective,’ because the waterfall does so in conjunction with reduced flow. When one increases flow in the face of a venous waterfall, P_RA_ necessarily rises.

## Data Availability

Not applicable.

## Abbreviations

P_MSF_: Mean systemic filling pressure
P_RA_: Right atrial pressure
R_VR_: Resistance to venous return
R_CARDIAC_: Cardiac resistance or cardiac function
OP: Operating point
P_PC_: Pericardial pressure
P_CRIT_: Critical closing pressure
Q_CIRCULATORY_: Blood flow
